# Drug repositioning of herbal compounds via a machine-learning approach

**DOI:** 10.1186/s12859-019-2811-8

**Published:** 2019-05-29

**Authors:** Eunyoung Kim, A-sol Choi, Hojung Nam

**Affiliations:** School of Electrical Engineering and Computer Science, Gwangju Institute of Science and Technology (GIST), Buk-gu, Gwangju, 61005 Republic of Korea

**Keywords:** Drug repositioning prediction, Machine learning, Data mining

## Abstract

**Background:**

Drug repositioning, also known as drug repurposing, defines new indications for existing drugs and can be used as an alternative to drug development. In recent years, the accumulation of large volumes of information related to drugs and diseases has led to the development of various computational approaches for drug repositioning. Although herbal medicines have had a great impact on current drug discovery, there are still a large number of herbal compounds that have no definite indications.

**Results:**

In the present study, we constructed a computational model to predict the unknown pharmacological effects of herbal compounds using machine learning techniques. Based on the assumption that similar diseases can be treated with similar drugs, we used four categories of drug-drug similarity (e.g., chemical structure, side-effects, gene ontology, and targets) and three categories of disease-disease similarity (e.g., phenotypes, human phenotype ontology, and gene ontology). Then, associations between drug and disease were predicted using the employed similarity features. The prediction models were constructed using classification algorithms, including logistic regression, random forest and support vector machine algorithms. Upon cross-validation, the random forest approach showed the best performance (AUC = 0.948) and also performed well in an external validation assessment using an unseen independent dataset (AUC = 0.828). Finally, the constructed model was applied to predict potential indications for existing drugs and herbal compounds. As a result, new indications for 20 existing drugs and 31 herbal compounds were predicted and validated using clinical trial data.

**Conclusions:**

The predicted results were validated manually confirming the performance and underlying mechanisms – for example, irinotecan as a treatment for neuroblastoma. From the prediction, herbal compounds were considered to be drug candidates for related diseases which is important to be further developed. The proposed prediction model can contribute to drug discovery by suggesting drug candidates from herbal compounds which have potentials but few were studied.

**Electronic supplementary material:**

The online version of this article (10.1186/s12859-019-2811-8) contains supplementary material, which is available to authorized users.

## Background

Over the past few years, it has become apparent that de novo drug discovery is a time-consuming and expensive process. Although expenditures for drug development have continued to increase, the number of approved or marketed drugs has stagnated [1, 2]. Drug repositioning, also known as drug repurposing, may be a viable alternative with regard to the productivity problem. The repositioning strategy reuses existing drugs for new indications. Because drugs that are currently on the market or have not been approved for reasons other than safety during clinical phases are used as candidates, drug repositioning presents the advantages of reducing the time and expenses associated with the overall pharmaceutical research and development process [3].

Most of the successful drug repositioning applied to date have relied on discovery by chance [4]. Therefore, systematic drug repositioning approaches are needed. Several computational methods for drug repositioning have been proposed, such as machine learning [5–8], network analysis [9, 10], and analysis of omics data [11–13].

Specifically, machine learning is a state-of-the-art screening technique that has attracted attention as a strategy for detecting potential indications. Therefore, drug repositioning could be converted to a supervised machine learning problem that predicts potential associations between marketed drugs and diseases. To predict novel drug indications, researchers in a previous study [7] constructed a prediction model using the classification algorithm of logistic regression. These authors utilized multiple similarities of drugs and disease properties as the features of machine learning. In another study [8], a feature-based drug repositioning approach was proposed. These authors used the phenotypic characteristics of drugs and the molecular characteristics of diseases to construct a prediction model to identify potential drug-disease associations. A support vector machine (SVM) algorithm was also employed to address the drug repositioning issue [5].

Meanwhile, natural products have been studied as the source of active ingredients in medicines. Among them, traditional herbal medicines including various natural compounds found in plants have been used for a long time [14, 15]. Herbal medicines are a highly promising source of new active compounds due to their low toxicity [14] and synergistic effects [16]. Despite various studies on drug repositioning, most of them have focused on predicting potential indications of existing drugs rather than those of herbal compounds. There have been several attempts to combine traditional herbal medicines with computational approaches; however, the overall number of computational approaches related to herbal medicines is limited, and such approaches tend to lag behind the state-of-the-art technology employed for these purposes [17–19]. To overcome these limitations, in the present study, we constructed several prediction models using various classification algorithms, and employed the constructed models to predict repositioning candidates among herbal compounds. We predicted new indications for herbs or herbal compounds using a computational model.

**Fig. 1:**
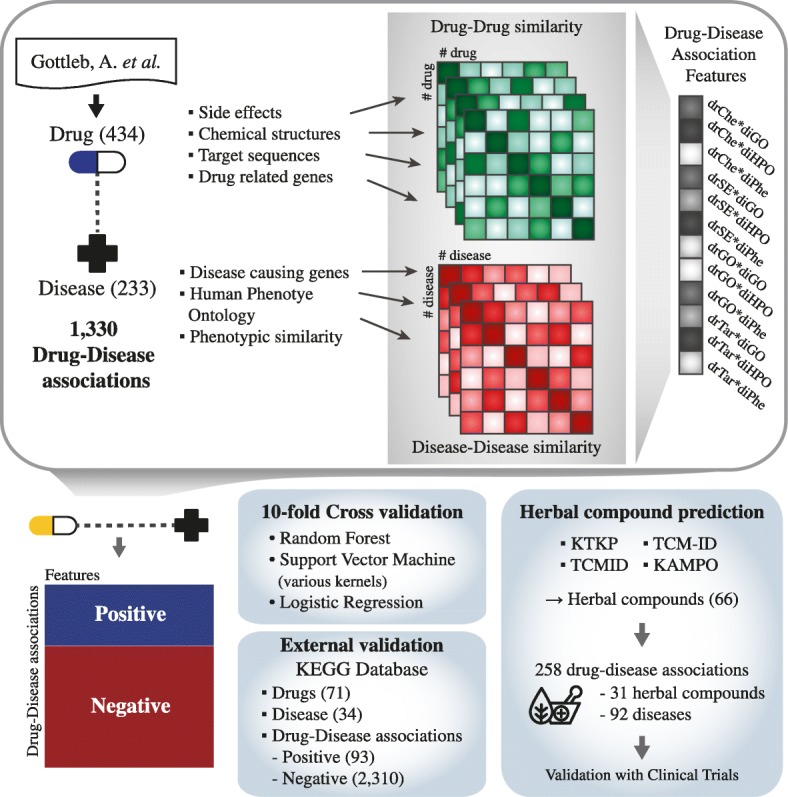
Overview of the proposed work. The training dataset was obtained from a previous study, and the drug and disease property data was retrieved from each database. Using the property information, similarity scores were calculated and combined to represent drug-disease associations. The drug-disease associations and similarity scores were used to construct a prediction model through cross-validation and external validation. Finally, the best performing model was applied to predict the repositioning candidates from the herbal compounds

The present study aims to predict new indications for existing drugs and additional herbal compounds based on a machine-learning approach. As shown in Fig. 1, firstly, we used reliable known drug-disease associations and obtained information on various properties of both drugs and diseases from different databases. We then calculated similarity scores for both drug and disease aspects based on their properties, and the similarity scores were employed as features in model construction. After data preprocessing, 1330 positive drug-disease associations were obtained. For negative associations, we randomly selected drug-disease pairs among all possible associations. Then for all training dataset, we calculated combined features of drug and disease similarities into a vector to represent drug-disease associations. Given the combinations of drug-disease similarities, prediction models were constructed using diverse classification algorithms, including both linear and nonlinear types. To evaluate the model, both linear and nonlinear classifiers were compared, after which we selected the prediction model that performed best for further use to predict new indications. Finally, we employed the constructed model to predict new associations between independent drugs and diseases and determined the capability of the model based on the predicted results. Moreover, new indications for herbal compounds stemming from various herbs were predicted as suggested candidates for drug repositioning.

## Methods

### Data preparation

For the purpose of drug repositioning, we used drug, disease, and drug-disease association data. The drug-disease associations to be employed as a training dataset were obtained from a previous study [7]. That study involved 1933 known drug-disease associations between 593 drugs from the DrugBank [19] database and 313 diseases registered in the Online Mendelian Inheritance in Man (OMIM) database [20].

### Similarity scores

In the present study, it was assumed that similar drugs are likely to serve as treatments for similar diseases; therefore, similarity scores were calculated for both drug and disease aspects. We calculated four types of drug-drug similarity and three types of disease-disease similarity and subsequently combined these scores to represent drug-disease associations.

#### Drug chemical structural similarity

Drugs with similar chemical structures are likely to serve as treatments for common diseases due to common therapeutic functions [5]. The degree of chemical similarity was calculated using information on chemical structures in the form of molecular fingerprints, which provide structural information by representing the presence or absence of substructures as binary digits. To obtain the molecular fingerprint data, we initially collected the canonical SMILES (Simplified Molecular-Input Line-Entry System) of 456 drugs from the DrugBank. The SMILES is a line notation describing the structures of compounds that can potentially be converted into fingerprints. We next obtained path-based fingerprints (referred to as FP2) providing structural information in 1021-bit vectors from Open Babel [21]. Finally, the chemical similarity scores between two drugs were computed using the Tanimoto coefficient [22], which equates to the Jaccard score [23].

#### Drug side-effect similarity

Information on side effects was retrieved from the SIDER [24] and OFFSIDES [25] databases. SIDER contains information on adverse drug reactions for marketed medicines, and OFFSIDES contains information on side effects that are not listed on the FDA’s official drug labels. We included the side effects that were common to both SIDER and OFFSIDES. Because both databases represent drugs showing side effects with STITCH [26] database IDs, ID mapping was necessary to link the drugs used here to their DrugBank IDs. To this end, the STITCH IDs were mapped to PubChem [27] CIDs (PubChem Compound Identifiers), and then converted from PubChem CIDs to DrugBank IDs using the compound ID mapping service UniChem [28]. As a result, information on 1844 drug side effects was obtained. We constructed binary vectors with a length of 1844, which represented whether a drug exhibits any side effects. Subsequently, the Jaccard score was employed to calculate the side effect similarity scores of the two drugs.

#### Drug target similarity

Target protein information for all drugs was provided by DrugBank, and the corresponding protein sequences were downloaded from the UniProt [29] database. Drug target similarity scores were computed based on the Smith-Waterman sequence alignment score [30] between the target proteins of two drugs. If two drugs have multiple targets, this method uses the maximum value of the target similarities of the two drugs.

#### Gene ontology (GO) similarity of drug-related genes

GO provides ontologies to annotate gene products. All drug-related genes are included in the DGIdb [31] database. We downloaded information on drug-gene interactions and extracted the Entrez gene IDs for the drugs of interest. The GO similarity score in each case was measured using the GoSemSim R package [32] based on the Resnik [33] method, which calculates the semantic similarities between two ontology terms. When drug-related genes exhibited multiple GO terms, the best-match average (BMA) combination strategy was used to combine semantic similarity scores.

#### Disease phenotypic similarity

We obtained disease phenotypic similarity scores using MimMiner [34], which calculates the similarities between the MeSH terms [35] of the diseases listed in OMIM.

#### Human phenotype ontology (HPO) similarity

HPO [36] is a standardized vocabulary describing phenotypic abnormalities in human diseases. The HPOSim R package [37] provides phenotypic similarity scoring based on HPO data for genes and diseases. The Resnik method was employed to measure the semantic similarities between HPO terms, and the BMA combination strategy was similarly used to combine similarities between diseases with multiple HPO terms.

#### GO similarity of disease-related genes

The GO semantic similarities of diseases were calculated using a method similar to that employed for drugs. A list of disease-related genes was provided by the DisGeNet [38] database. We mapped the OMIM IDs of diseases to UMLS Concept Unique Identifiers (CUIs) [39]. Using these UMLS CUIs, we obtained information concerning disease-gene associations from DisGeNet. The process of calculating the degrees of GO similarity was identical to the method used for drugs.

### Gold standard dataset

After the retrieval of feature information, the final training dataset, which is the gold-standard dataset, was established. Due to data limitations, 159 drugs were excluded because they did not have available structure or side-effect information. Similarly, 80 diseases with no information pertaining to disease-related genes and known associations with gold-standard drugs were removed. Finally, we utilized 1330 known drug-disease associations encompassing 434 drugs and 233 diseases as the gold-standard dataset. Moreover, we selected random pairs of drugs and diseases that were not included in the positive set as the negative set. Due to the imbalance between positives and negatives, a random under-sampling method was adopted to obtain a 1:2 ratio of positives to negatives.

### Classification features for drug-disease associations

**Fig. 2:**
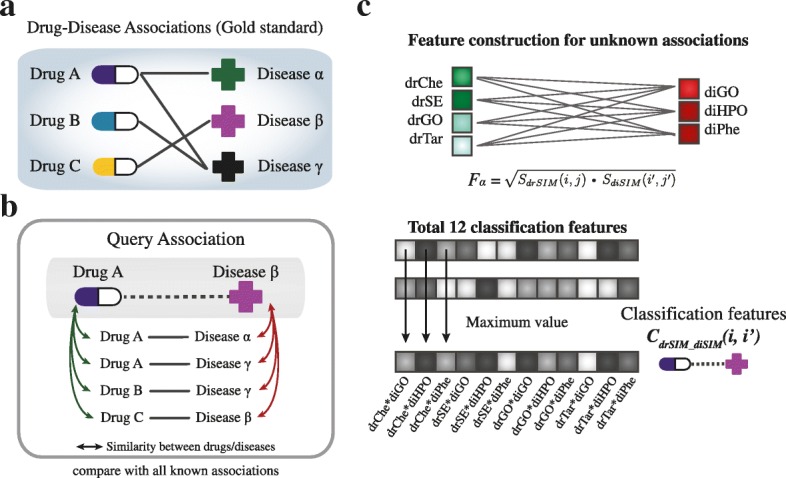
Calculation of classification features for drug-disease associations. **a** An example of drug-disease associations considered as gold standard. **b** From known drug-disease associations, classification features were calculated using the similarity scores between the drugs and diseases of each association type. **c** Then, similarity scores of each association were combined into a Cartesian product, resulting in a total of 12 features, and the maximum value was selected to represent the query association

To represent drug-disease associations, we employed a previously described method for constructing classification features [7]. The combination of the four types of drug-drug similarity and three types of disease-disease similarity constituted the classification features, as a way to express drug-disease associations. Figure 2 represents the process of feature calculation. Given known drug-disease associations from gold standard dataset (A), we calculated combined features by comparing properties in terms of drugs and diseases to those of known pairs. As shown in the Fig. 2 (B), the association between drug A and disease β is not known. To represent the association, we compared the query association with four known associations. To construct combination features, we first calculated each drug similarity and disease similarity. For each combination, four types of drug-drug similarity and three types of disease-disease similarity were combined using geometric mean (C) [40]. In the Fig. 2, *S*_*drSIM*_ represents drug similarities, consists of *S*_*drChe*_(*i*, *j*), *S*_*drSE*_, *S*_*drGO*_, and *S*_*drTar*_ which each represents the structural, side-effect, GO, and target sequence similarity between drugs *i* and *j*. For disease similarity *S*_*diSIM*_, *S*_*diGO*_(*i*^′^, *j*^′^) represents the GO similarity between diseases *i*^′^ and *j*^′^. Similarly, *S*_*diHPO*_ and *S*_*diPhe*_ represent each HPO and phenotypic similarity, respectively. For drug *i* and disease *i*^′^ we calculated combined similarity feature vector *F*_*α*_ using Eq. (1) for all known associations, *M* and *N* represent the number of drugs and diseases in known associations.

1 $$ {\displaystyle \begin{array}{c}{F}_{\alpha }=\sqrt{S_{drSIM}\left(i,j\right)\cdot {S}_{diSIM}\left({i}^{\hbox{'}},{j}^{\hbox{'}}\right)},\\ {} where\ j=1,2\dots, M,{j}^{\hbox{'}}=1,2,\dots, N\end{array}} $$

This process was conducted for all selected known associations.

Then, maximum values of each index of feature vectors were used to represent the pair of drug *i* and disease *i*^′^. Here, we combined the maximum values because the more similar the two associations are, the larger the combined value becomes, suggesting that the query association likely represents a potential association. In other words, final classification features indicate how similar the query association is to the entire known association dataset. Moreover, we constructed combined features with mean values to compare the performance which under consideration of overall similarity with known associations.

### Model construction

We constructed a prediction model that predicts whether particular drug-disease associations present potential using both linear and nonlinear classification algorithms. Although most previous studies [7, 8] have employed a logistic regression algorithm, the distribution of the gold-standard dataset plotted using the t-SNE algorithm [41] showed that the shape of the data was nonlinear (Additional file 1: Figure S1) – the distribution seems like the dataset can be classified by linear models, but better with nonlinear classifiers. Therefore, we constructed prediction models with linear classification as well as nonlinear algorithms. In the case of linear classification models, logistic regression and SVM with a linear kernel were employed. Additionally, for the nonlinear classification algorithms, random forest and SVM with nonlinear kernels of a radial basis function (RBF) and a polynomial were used. The performance of each prediction model was measured via 10-fold cross-validation and external validation.

### Independent test dataset

For external validation, we employed an independent test dataset sourced from the literature [9]. This study provided 144 new associations for 115 drugs collected from KEGG [42], and the associations did not overlap with the gold-standard associations. The independent test dataset was also preprocessed as the training dataset. We removed 44 drugs from the set of original drugs due to missing chemical structures or missing information on side-effects. Consequently, the final independent test dataset contained 89 associations between 71 drugs and 34 diseases.

### Herbal compound dataset

Data on herbal compounds were sourced from multiple databases. First, we obtained the herb entries from the Korea Traditional Knowledge Portal (KTKP, http://www.koreantk.com), the Traditional Chinese Medicine Integrated Database (TCMID) [43], the Traditional Chinese Medicine Information Database (TCM-ID) [44] and the Japanese Traditional Medicine and Therapeutics (KAMPO) database [45]. We then gathered information about the herb-compound and herb-phenotype associations from the KTKP, TCM-ID, and TCMID. In addition, information on side effects was obtained from SIDER, and gene information related to the herbal compounds were collected from the BindingDB [46], MATADOR [45] and STITCH databases. Information on the chemical structure of herbal compounds was sourced from DrugBank. Finally, we preprocessed the dataset of 66 herbal compounds and calculated the similarity scores between the herbal compound dataset and the training dataset.

## Results

### Our constructed prediction model shows more than 90% accuracy

**Fig. 3:**
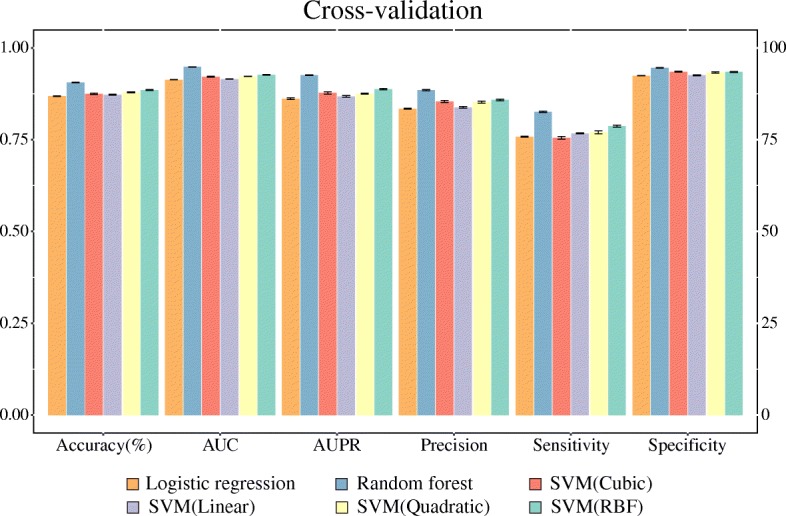
Performance of prediction models in cross-validation. Overall, the model involving the random forest algorithm performed better than those using other algorithms

**Fig. 4:**
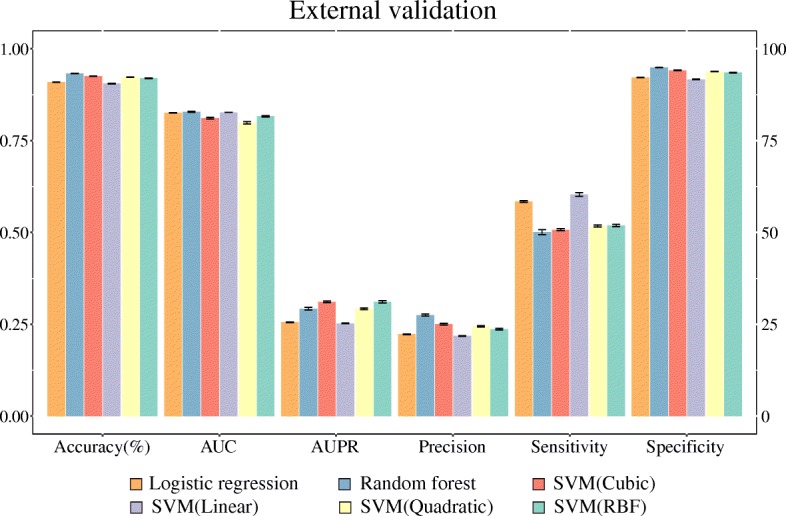
Performance of prediction models in external validation. The random forest model showed the best performance in terms of accuracy and AUC

We attempted 10-fold cross-validation to construct drug-disease association prediction model by means of under-sampling 30 times independently for each prediction model. Figure 3 and Additional file 2: Table S1 show the evaluation results of the prediction models trained by each classification algorithm. Here, we calculated and reported performances by six evaluation metrics – accuracy, AUC, AUPR, sensitivity, specificity, and precision. Upon cross-validation, the random forest method resulted in the highest accuracy levels and AUC values among all classifiers (Accuracy = 90.6%, AUC = 0.948), followed by SVM (RBF), SVM (Quadratic), and SVM (Cubic). The linear classifiers of logistic regression and SVM (Linear) showed the poorest performance. We also performed independent tests for external validation. Upon external validation, the random forest method resulted in better performance than the other classifiers in terms of both accuracy and the AUC. The accuracy showed the following decreasing order: SVM (Quadratic), SVM (Cubic), SVM (RBF), logistic regression, SVM (Linear). With regard to AUC, unlike accuracy, the linear classifiers logistic regression and SVM (Linear) showed better performance than the nonlinear classifiers, except for the random forest method. Detailed information on performance is presented in Fig. 4 and Additional file 2: Table S2. Moreover, the performance of random forest model with features of mean values are compared in Additional file 1: Figure S2.

In addition, we further evaluated the prediction model by training only with drugs included in the test set to validate the ability to predict drug repositioning candidates. We filtered 168 positive and 9154 negative associations among the total training dataset, representing the associations related to 71 drugs in the test dataset. Using the filtered dataset, an additional prediction model with sampling of negative associations was constructed using the random forest method, which performed the best. Then, the model was validated with the independent test dataset. In this validation, the same drugs were included in the two datasets, but with different indications. The differences resulted in different drug-disease associations and, thus, drug repositioning (i.e., providing new indications for a given drug). The model performance under the described conditions is represented in Additional file 1: Figure S3. Although overall performance decreased slightly, by one to 4 %, compared with external validation using the whole dataset, reasonable results were still obtained. This result shows that our prediction model predicts unknown associations among both known and unknown drugs and the performances were both high despite of small dataset.

**Fig. 5:**
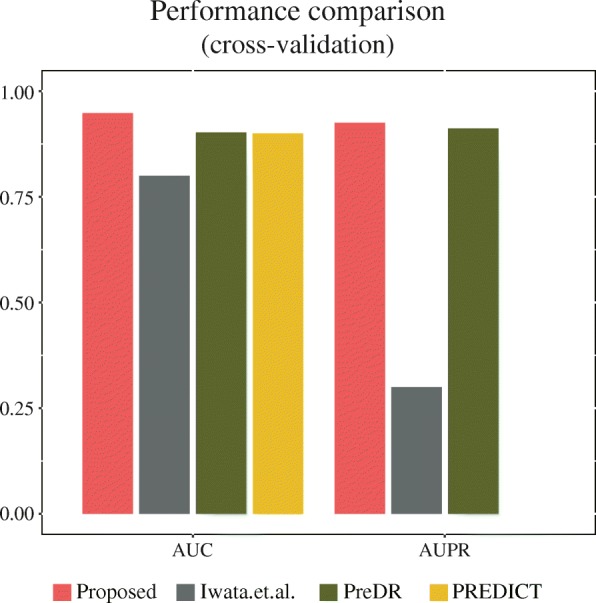
Comparison of performance with previous studies. The performance of the constructed model with the random forest algorithm was compared with related studies. The AUC and AUPR metrics were used for the comparison, as previously reported

Finally, we compared the constructed model with previous studies. Figure 5 shows the comparison of performance with three related studies. Here, we compared the performances in terms of the AUC and AUPR values obtained from each of the previous studies. The performance of PREDICT and PreDR was evaluated using similar dataset, since we obtained the gold-standard dataset from these two sources. Here, the number of dataset is different each other because the feature information used for each model is different. The differences from previous models are the feature information used, so the combined features are also different that might be the factor of performance increase. As the authors only reported cross-validation results, we could not compare the external validation performance. As shown in Fig. 5, the present model performed better than both the PREDICT and PreDR in terms of the AUC values, which were 0.9 and 0.908, respectively. The performance was further compared in terms of the AUPR since the dataset used is imbalanced and the negative dataset is not true negative associations, but is instead unknown associations. The AUPR of our model was slightly better compared to that of the PreDR (AUPR = 0.912) and there was no AUPR for PREDICT. However, the model of Iwata et al. was constructed with a different dataset from KEGG, so this comparison might not be precise. Nevertheless, our new model showed better performance, especially in AUPR.

### Potential new indications for existing drugs predicted using the present model

Using the prediction model trained by the random forest algorithm, which showed the best performance, we predicted potential drug-disease associations in the independent test dataset. We then extracted the drug-disease associations that were predicted to have associations but had no known associations in the gold standard dataset across the trials. Although the true label was negative, predicting the association as positive may indicate that potential associations are still unknown. As a consequence, 37 associations between 20 drugs and 18 diseases were predicted among 2310 associations. For validation, we manually queried predicted potential associations based on clinical trials (https://clinicaltrials.gov/). Because one OMIM disease term could include several disease concepts, the OMIM disease names were mapped to multiple numbers of UMLS concepts and then queried. The results are given in Table 1**,** which lists the associations that showed clear evidence in the manual search.

**Table 1:** Predicted indications for existing drugs

Drug	DrugBank ID	Predicted Indications	Predicted Probability	ClinicalTrials.gov Identifier
Bromocriptine	DB01200	Parkinson disease	0.9879	NCT01673724
Methylprednisolone	DB00959	Osteoarthritis	0.9403	NCT00805519
		Autoimmune hemolytic anemia	0.9328	NCT01134432
		Acute myeloid leukemia	0.8214	NCT00309907
Triamcinolone	DB00620	Osteoarthritis	0.8421	NCT02295189

Bromocriptine was predicted to serve as a treatment for Parkinson’s disease by the present model, with markedly high probability scores, and we found several related completed studies among clinical trials. These experiments addressed the safety and efficacy of pramipexole and bromocriptine in Parkinson’s disease patients in phase three or four. Methylprednisolone was predicted for potential repositioning to three different diseases: osteoarthritis, autoimmune hemolytic anemia, and acute myeloid leukemia (AML). These associations were not precisely matched in clinical trials but are somehow related. Methylprednisolone has been experimentally tested for the treatment of knee osteoarthritis. Additionally, prednisolone was tested with Rituximab for the treatment of warm-antibody-dependent autoimmune hemolytic anemia, for which the conventional treatment is a high-dose glucocorticoid. Prednisolone is also used to treat AML patients for idiopathic pneumonia syndrome after stem cell transplantation. Finally, the triamcinolone-osteoarthritis association resulted in a number of studies involving several types of osteoarthritis, confirming that the present model showed good performance in terms of prediction. This clinical evidence shows the predictive power of the constructed model, and other detailed predicted results are listed in Additional file 3: Table S3.

### The constructed prediction model provides potential new indications for natural herbal compounds

Finally, we applied the prediction model to herbal compounds to infer new indications for herbal compounds. Herbal compounds have attracted attention as candidates for drug development due to their low side-effects and stability, and several previous studies have been performed to predict the potential of herbal compounds through computational models. Therefore, we predicted potential new indications for natural herbal compounds using the constructed model. Among the associations between 66 herbal compounds and 233 diseases, we excluded compounds and diseases having associations in the gold standard dataset; thus, finally, 258 associations between 31 herbal compounds and 92 diseases were predicted. The same process identifier conversion from OMIM to UMLS was performed. Then, we searched for the predicted associations between herbal compounds and diseases in clinical trials, consistent with the method employed for existing drugs. Table 2 lists the predicted results with prediction probabilities.

**Table 2:** Predicted indications for herbal compounds

Compound	Disease	Predicted Probability	ClinicalTrials.gov Identifier
Testosterone	Calcification	0.9245	NCT00838838
	Polycystic ovary syndrome	0.8584	NCT00757185
	Hyperplasia	0.6345	NCT00194675
Cortisol	Edema	0.9067	NCT00820092
	Alopecia	0.7454	NCT01453686
Ephedrine	Headache	0.8883	NCT00378144
	Cough	0.8836	NCT00378144
Podophyllotoxin	Leukemia	0.8141	NCT01260714
(−)-Prostaglandin E1	Hypertension	0.7911	NCT01467076
Irinotecan	Neuroblastoma	0.6746	NCT00644696
Salicylic acid	Hypertension	0.6071	NCT01741922

Testosterone, which is included in many herbs, was predicted for several diseases. Among these diseases, testosterone gel has been tested for shrinking large prostate glands in several studies. Ephedrine (specifically pseudoephedrine) has been experimentally tested regarding its efficacy and safety for the treatment of the common cold. Although not specifically examined in experiments, podophyllotoxin has been tested with several drugs, including etoposide. Podophyllotoxin and its derivatives are precursors of anti-tumor agents such as etoposide, which was the tested drug. This result shows that our model can predict more complex associations based on a combination of features. Moreover, irinotecan was predicted to serve as a treatment for neuroblastoma, and several matching studies were found among clinical trials. Experiments involving irinotecan have been performed in combination with chemotherapy to stop the growth of neuroblastoma cells. According to these results, repositioning candidates predicted from herbal compounds have been examined in clinical trials, indicating that the constructed model is reliable. Moreover, other predicted associations could represent potential repositioning candidates. The detailed prediction results are listed in Additional file 3: Table S4.

Furthermore, we analyzed which specific similarities are related to predicting drug-disease associations. Among 258 associations, we filtered 50 drug-disease pairs that showed highest match score when searched on PubMed. Then, we traced back each similarity score of 12 herbal compounds in the selected associations. We focused on drug similarities first since large number of phenotypic similarities showed zeros and ones that might be resulted from calculating ontologies with disease related identifiers. Interestingly, drugs showed slightly different patterns in similarities. Each type of similarity scores varies depending on the drug – salicylic acid showed low similarity scores of chemical structure and GO (0.623 and 0.626 for each), but atropine showed high score in GO (0.937) and still low score in structure (0.674). More specifically, podophyllotoxin showed different similarity scores in each type – side effect (0.985), target sequence (1), GO (0.445), and structure (0.760). Then, we extracted 18 related diseases of each similar drug in known associations to compare with predicted indication which is ‘Dohle bodies and leukemia’. Overall similarity scores are low considering their values of *S*_*diPhe*_ (0.145), *S*_*diGO*_ (0.348), and *S*_*diHPO*_ (0.268), but it showed high similarities in maximum value aspect except the GO similarity which score was 0.63 the highest. From this result, we can infer that our combined features may reflect information of both drug and disease properties and each property type, so that model can predict associations that includes low similarity from one aspect.

## Discussion

Drug repositioning plays a key role in drug development, and systematic computational approaches could be promising for achieving this goal. Many computational drug-repositioning methods have been proposed using state-of-the-art techniques, such as machine learning supported by large volumes of omics data. Machine learning approaches consider both drug and disease characteristics into prediction models resulting in higher performance. Also, we can analyze important features in the model. However, the complexity and relative scarcity of drug-disease association data can influence the performance capability of the applied model. Additionally, previous studies have not shown practical applications beyond predicting potential indications for existing drugs. Thus, in the present study we constructed a prediction model based on the properties of drug-disease associations and applied the model for prediction of repositioning candidates in an herbal compound dataset.

First, we attempted to construct prediction models with several classification algorithms. Given that the distribution of the drug-disease associations was nonlinear, we employed both linear and nonlinear classification algorithms during model construction. Using internal validation and external validation, we confirmed that the performance of nonlinear classifiers (the random forest algorithm and SVM with a nonlinear kernel) was superior to that of linear classifiers (logistic regression and SVM with a linear kernel). Upon cross-validation, the accuracy and AUC values of the nonlinear classifiers were higher than those of the linear classifiers, with the random forest method showing the best performance. Upon external validation, the random forest method also achieved the highest accuracy and AUC values. These results suggested that the random forest algorithm is suitable for use in a prediction model for drug repositioning and can be applied for predicting repositioning candidates among herbal compounds.

Using the prediction model trained by the random forest algorithm, we made predictions based on an independent test dataset. The drug-disease associations predicted as false positives were filtered to detect associations with a high likelihood of repositioning. The selected drug-disease associations were validated through manual searches of clinical trials. Based on these results, we identified potential candidates and assessed the potential of the prediction model for herbal compound prediction.

Finally, potential indications for herbal compounds were inferred in addition to the prediction of indications for existing drugs. Analysis of the specific ingredients of herbs could help to develop various uses for the herbs. Such analyses may provide evidence of the effectiveness of an herb, in addition to suggesting potential candidate herbal medicines.

## Conclusions

In this study, we introduced a prediction model for drug repositioning based on a similarity-based assumption. We collected and preprocessed three datasets, which included the properties of drugs, diseases, and drug-disease associations. We then calculated classification features using multiple similarity measures to express the drug-disease associations. Based on these classification features, we constructed prediction models, which were trained using linear classifiers as well as nonlinear classifiers. Through both internal validation and external validation, we assessed the performance of each model and found that nonlinear classifiers, particularly the random forest method, outperformed linear classifiers. The prediction model trained via the random forest method was applied to an independent test dataset and an herbal compound dataset to predict potential drug-disease associations. In the independent test, the accuracy of the model was above 90% and resulting false positive associations were considered repositioning candidates to be further validated. Moreover, the model was applied to predict associations between herbal compounds and diseases. The predicted repositioning indications for existing drugs and herbal compounds were manually validated with clinical trial results, and the results showed that herbal compounds could serve as drug candidates for corresponding diseases. This finding is important because the mechanisms and usage of herbal compounds are not well understood, despite their potential as drug candidates. Therefore, the proposed prediction models can contribute to drug discovery in terms of the drug repositioning of herbal compounds by indicating their potentialities for different diseases.

Although the proposed method showed an outstanding performance, the method still has room to be improved. First, the amount of available data is limited to the results of data retrieval for features. Because not all feature information is available for all drugs and diseases, a certain amount of data should be excluded, which can decrease performance when the machine-learning approach is employed. Similarly, information on herbal compounds is limited. Second, the negative dataset was randomly selected, indicating the potential for false positives (i.e., not true negatives). This characteristic of a negative dataset can cause confusion during the training of prediction models. This problem can be solved using more precise data labels to improve prediction models. Lastly, there is a fundamental problem with similarity-based methods, in that these strategies do not work under certain conditions, such as when drugs are macroscopically dissimilar but share key substructures at the detailed level. Regarding this point, feature-based methods may be better than similarity-based methods, including the neural network approach.

## Additional files

Additional file 1: **Figure S1.** Data distribution of the training dataset. **Figure S2.** Performance comparison in cross-validation with different feature aggregation method. **Figure S3.** Prediction ability for drug repositioning. (PDF 303 kb)

Additional file 2: **Table S1.** Performance of prediction models in cross-validation. **Table S2.** Performance of prediction models in external validation. (PDF 51 kb)

Additional file 3: **Table S3.** Detailed prediction results of existing drugs. **Table S4.** Detailed prediction results of herbal compounds. (XLSX 15 kb)

## Abbreviations

AML: Acute myeloid leukemia
AUC: Area under curve
AUPR: Area under precision-recall curve
BMA: Best-match average
GO: Gene ontology
HPO: Human phenotype ontology
RBF: Radial basis
SMILES: Simplified molecular-input line-entry system
SVM: Support vector machine function
t-SNE: t-distributed stochastic neighbor embedding
UMLS: Unified Medical Language System
