# Supplementary material for: Drug repositioning of herbal compounds via a machine-learning approach
Source: BMC Bioinformatics. 2019 May 29;20(Suppl 10):247. doi: 10.1186/s12859-019-2811-8 (PMC6538545; doi:10.1186/s12859-019-2811-8)
Supplement: Supplementary file 1 — Figure S1. Data distribution of the training dataset. Figure S2. Performance comparison in cross-validation with different feature aggregation method. Figure S3. Prediction ability for drug repositioning. (PDF 303 kb) [file 12859_2019_2811_MOESM1_ESM.pdf]

Figure S1 – Data distribution of training dataset

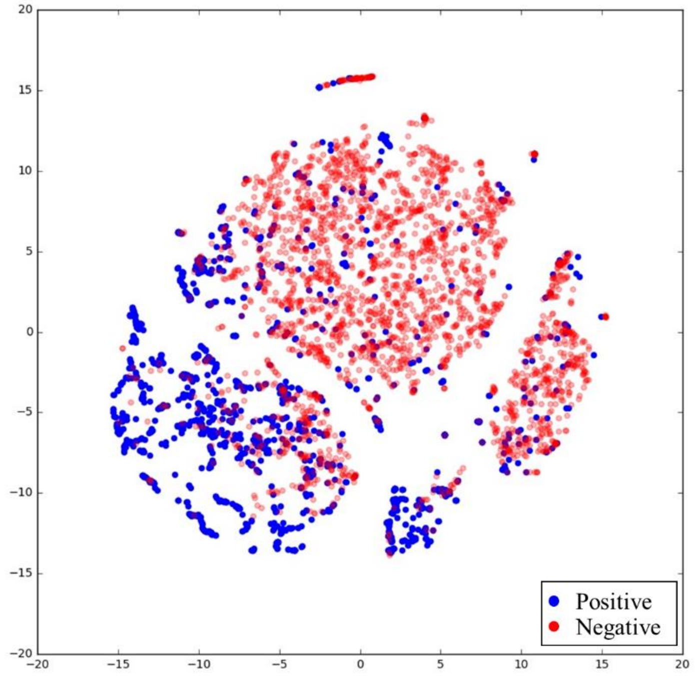

Blue points represent the positive class and red points represent the negative. The distribution was plotted using t-SNE algorithm.

Figure S2 – Performance comparison in cross-validation using different features

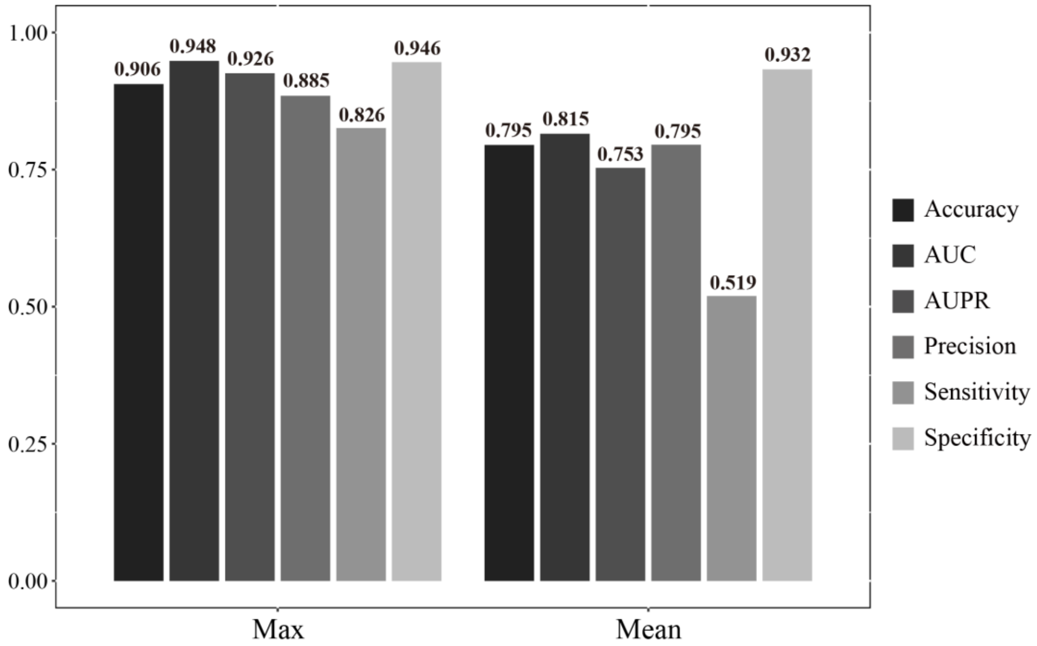

Performances of prediction models constructed with features obtained by each maximum and mean values. Models were trained with random forest algorithm under same condition.

Prediction model with features from maximum values outperformed and applied to further evaluation.

Figure S3 – Prediction ability for drug repositioning

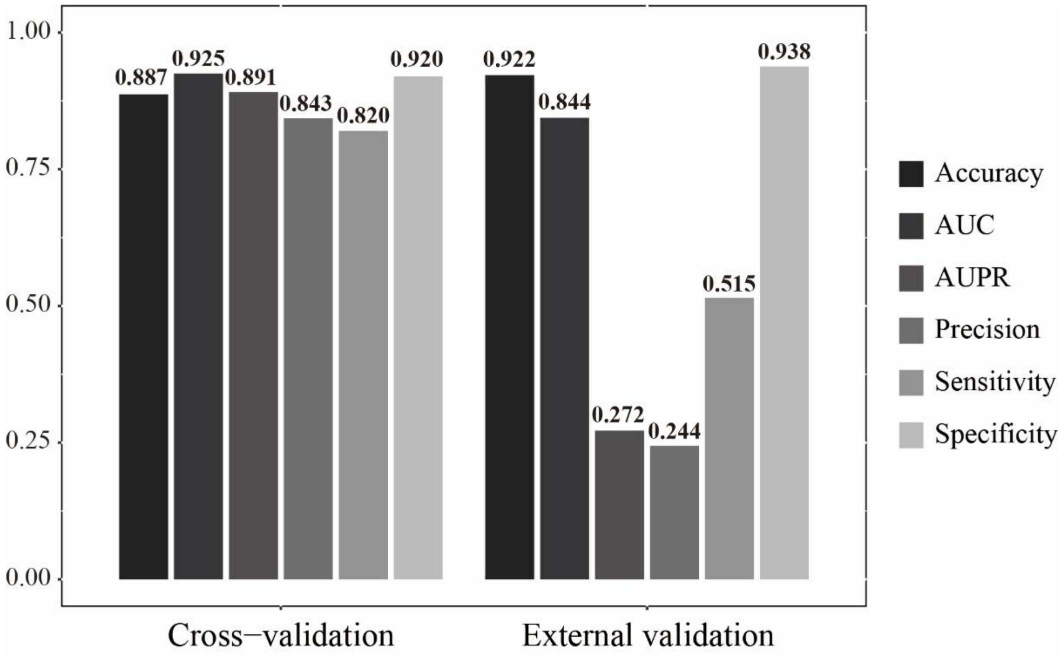

Prediction model performance trained with only drugs in the test set. Constructed model showed similar performance compared to models with entire dataset.
