# Supplementary material for: Drug repositioning of herbal compounds via a machine-learning approach
Source: BMC Bioinformatics. 2019 May 29;20(Suppl 10):247. doi: 10.1186/s12859-019-2811-8 (PMC6538545; doi:10.1186/s12859-019-2811-8)
Supplement: Supplementary file 2 — Table S1. Performance of prediction models in cross-validation. Table S2. Performance of prediction models in external validation. (PDF 51 kb) [file 12859_2019_2811_MOESM2_ESM.pdf]

Table S1 – Performance of prediction models in cross-validation

	**Logistic regression**	**Random forest**	**SVM (Linear)**	**SVM (Quadratic)**	**SVM (Cubic)**	**SVM (RBF)**
Accuracy (%)	86.8797 (± 0.0009)	90.5865 (± 0.0009)	87.253 (± 0.0012)	87.8546 (± 0.0013)	87.4962 (± 0.0016)	88.5188 (± 0.0012)
AUC	0.9131 (± 0.006)	0.9482 (± 0.0006)	0.9151 (± 0.0007)	0.9222 (± 0.0005)	0.9213 (± 0.0011)	0.9267 (± 0.0012)
AUPR	0.8616 (± 0.0022)	0.9256 (± 0.0008)	0.8678 (± 0.0023)	0.8752 (± 0.0017)	0.8775 (± 0.0031)	0.8874 (± 0.0020)
Sensitivity	0.7580 (± 0.0016)	0.8257 (± 0.0017)	0.7672 (± 0.0017)	0.7703 (± 0.0040)	0.7551 (± 0.0040)	0.7865 (± 0.0027)
Specificity	0.9242 (± 0.0008)	0.9459 (± 0.0009)	0.9252 (± 0.0012)	0.9327 (± 0.0018)	0.9349 (± 0.0013)	0.9345 (± 0.0014)
Precision	0.8342 (± 0.0016)	0.8846 (± 0.0018)	0.8378 (± 0.0024)	0.8520 (± 0.0031)	0.8538 (± 0.0028)	0.8581 (± 0.0026)

Table S2 – Performance of prediction models in external validation

	**Logistic regression**	**Random forest**	**SVM (Linear)**	**SVM (Quadratic)**	**SVM (Cubic)**	**SVM (RBF)**
Accuracy (%)	90.8962 (± 0.0005)	93.2541 (± 0.0005)	90.5030 (± 0.0005)	92.2676 (± 0.0006)	92.5163 (± 0.0006)	91.9730 (± 0.0008)
AUC	0.8255 (± 0.0003)	0.8285 (± 0.0013)	0.8268 (± 0.0003)	0.7986 (± 0.0033)	0.8114 (± 0.0021)	0.8162 (± 0.0021)
AUPR	0.2553 (± 0.0006)	0.2924 (± 0.0033)	0.2525 (± 0.0007)	0.2922 (± 0.0024)	0.3112 (± 0.0023)	0.3110 (± 0.0033)
Sensitivity	0.5843 (± 0.0022)	0.5004 (± 0.0069)	0.6034 (± 0.0049)	0.5176 (± 0.0027)	0.5075 (± 0.0028)	0.5191 (± 0.0030)
Specificity	0.9215 (± 0.0005)	0.9492 (± 0.0006)	0.9167 (± 0.0007)	0.9383 (± 0.0007)	0.9413 (± 0.0007)	0.9352 (± 0.0009)
Precision	0.2229 (± 0.0009)	0.2751 (± 0.0025)	0.2181 (± 0.0009)	0.2446 (± 0.0018)	0.2502 (± 0.0019)	0.2364 (± 0.0023)
